# The effect of mindfulness meditation on dental anxiety during implant surgery: a randomized controlled clinical trial

**DOI:** 10.1038/s41598-023-49092-3

**Published:** 2023-12-07

**Authors:** Onur Ucak Turer, Mustafa Ozcan, Bahar Alkaya, Furkan Demirbilek, Nilgun Alpay, Gulcin Daglioglu, Gulsah Seydaoglu, M. Cenk Haytac

**Affiliations:** 1 Department of Periodontology, Faculty of Dentistry, Cukurova University, Adana, Turkey; 2 Department of Anesthesiology and Reanimation, Faculty of Dentistry, Cukurova University, Adana, Turkey; 3 Department of Medical Biochemistry, Faculty of Medicine, Cukurova University, Adana, Turkey; 4 Department of Biostatistics, Faculty of Medicine, Cukurova University, Adana, Turkey

**Keywords:** Psychology, Medical research

## Abstract

Dental implant surgery is almost always associated with patient anxiety. Anxiety during dental surgical procedures triggers an increase in sympathetic activity. Mindfulness meditation (MM) is often associated with high levels of relaxation in the form of increased parasympathetic tone and decreased sympathetic activity. However, the effect of MM on dental anxiety is not clear. The current study aimed to show the effects of a MM as a sedative technique during dental implant surgery by examining the State-Trait Anxiety Inventory (STAI-S), bispectral index (BIS), cortisol levels (CL), systolic (SBP) and diastolic blood pressure (DBP), heart rate (HR) and saturation (SpO_2_) parameters. HR, SBP, DBP, SpO_2_, BIS score and CLs were compared at the baseline, immediately before-, during-, and immediately after surgery between the test and control groups. We found that the MM resulted in significant decrease in BIS together with positive effects on hemodynamic parameters (decrease of HR, SBP, DBP and increase of SpO_2_), psychological findings (improvement on STAI-S scores) and biochemical outcomes (decreased CL). In conclusion, the results demonstrate that MM appeared to be a reliable strategy for managing stress during dental implant operation with benefits in psychological, physiological and biochemical outcomes.

## Introduction

Dental implant surgery often lasts 1 to 2 h and is almost always associated with patient anxiety and psychological stress. The term “stress” describes the effects of psychosocial and environmental factors on physical and/or mental well-being^1–6^. The physiology of the stress response includes fast and slow components. A fast response results in increased secretion of norepinephrine and epinephrine from the adrenal medulla into the circulation leading to increased blood pressure, heart rate, cardiac output, behavioral activation (alertness, vigilance) and this pathway is regulated by the sympathetic-adreno-medullar (SAM) axis. A slow response, mediated by the hypothalamic pituitary adrenal (HPA) axis results in the release of corticotrophin-releasing hormone into the circulation and eventually adrenocorticotrophin hormone (ACTH) into the bloodstream. ACTH stimulates the adrenal cortex to secrete glucocorticoid hormones, such as cortisol into the circulation. The SAM pathway is the route through which the brain directs the sympathetic branch of the autonomic nervous system (ANS) to activate in response to short-term stress^7–9^ The ANS plays a key role in stress reactivity via its two main divisions, i.e. the Sympathetic Nervous System (SNS) and the Parasympathetic Nervous System (PNS)^10^.

Acute stress and anxiety during dental surgical procedures trigger an increase in sympathetic activity that is particularly undesirable in patients with cardiovascular diseases^3^. Therefore, intravenous sedation has been reported as clinically useful in preventing sympathetic hyperactivity^3,11–15^. Although the ideal sedation agent used in dental surgery should provide a rapid onset of action and easily reversible, there have always been some serious potential risks associated with sedation such as airway obstruction^16^, hemodynamic changes^17^, amnesia effect^18,19^, bradycardia, agitated behavior^15^. Dental anxiety can also be managed by non-pharmacological anxiety alleviation strategies including behaviorally or cognitively oriented psychotherapeutic interventions such as relaxation techniques, guided imagery, hypnotherapy, acupuncture, cognitive behavioral therapy^20^.

In current research contexts, the mindfulness meditation (MM) technique is defined as nonjudgmental attention to experiences in the present moment^21,22^. Mindfulness is practiced to achieve enduring happiness and to gain insight into the view of the true nature of existence^21,23^. Bishop et al. (2004) suggested a two-component model of mindfulness, where the first component is the regulation of attention, and the second component involves approaching one’s experiences with an orientation of curiosity, openness, and acceptance^21,24^. The practice of MM encompasses focusing attention on the experience of thoughts, emotions, and body sensations, simply observing them as they arise and pass away resulting in body awareness, attention regulation, emotion regulation and change in perspective on the self^22^. Body awareness is considered an integral part of the mindfulness construct and it has been proposed as one of the major mechanisms of mindfulness interventions^25–27^.

In the medical and psychological research, MM has been reported to produce beneficial effects on a number of psychiatric and stress-related symptoms and has therefore increasingly been incorporated into psychotherapeutic programs^28,29^ including anxiety^30,31^, depression^30,32^ and chronic pain^33^ management. Furthermore, MM positively influences aspects of physical health (i.e. improved immune function^34,35^ reduced BP and cortisol levels^34^) and psychological well-being^36,37^ and cognitive functioning^38^.

MM is often associated with high levels of relaxation in the form of increased parasympathetic tone and decreased sympathetic activity^39^. Peripheral physiological changes have been observed with some consistency, including decreased HR^40^, decreased BP^41^ and decreased cortisol levels^34^. Cortisol is the end product of HPA axis functioning and salivary cortisol is a commonly used measure of physiological stress responses and provides information about cortisol levels and changes over periods of time^22,42,43^.

Mindfulness-based techniques have been suggested to be useful for managing dental anxiety including implant surgery^44,45^. However, previous studies on the relationship between mindfulness and dental anxiety only included trait measures of dental anxiety and the results have been inconsistent and limited to specific samples and have not investigated possible mechanisms that may be involved in the relationship between mindfulness and dental anxiety^44,46,47^.

We hypothesized that patients randomly assigned to receive pre-operative mindfulness meditation would show a greater improvement in psychological distress and the vital signs than non-meditated patients. Therefore, the aim of the current study was to evaluate the effects of a brief mindfulness meditation (short body scan) as a sedative technique during dental implant surgery via examining the subjective (State-Trait Anxiety Inventory) and objective (BIS, cortisol levels, SBP, DBP, HR, SpO_2_) parameters.

## Methods

### Study design

The study was designed as a randomized controlled clinical trial with two parallel groups and a single-blinded approach, performed at the Periodontology Department of Cukurova University, Faculty of Dentistry (Adana, Turkey) between December 2021 and August 2022. The study protocol was approved by the Institutional Ethical Committee (03.06.2022 123/53), registered on ClinicalTrial.gov (NCT05748223-Feb 28, 2023) and performed in accordance with the Helsinki Declaration of Human Studies. Before the procedure, the patients were given full and adequate verbal and written information about the purpose of the study and written informed consent was obtained.

### Study participants

Individuals were eligible to participate and included in the study if they were:

- Age between 25 and 65 years old,
- Single tooth loss in the maxillary premolar–molar region with partial bone healing (type 3) or fully healed ridge type 4,
- Sufficient bony ridge and keratinized tissue for implant placement without the need for simultaneous bone grafting or sinus lift procedures,
- No signs of acute oral infection,
- Periodontally and systemically healthy.

The exclusion criteria were:

- Diabetes mellitus,
- Smoking,
- Pregnancy,
- Diagnosis of anxiety disorders/psychiatric disorder/depression and whether or not they were seeking any form of treatment for these conditions,
- History of radiation therapy at head and neck area and chemotherapy.

### Randomization, allocation concealment, assignment of interventions and blinding

Patients were assigned to one of the two treatment groups with the use of computer-generated randomization table (test group: mindfulness meditation (MM); control group: non-meditated. Treatment assignment was concealed to the meditator (O.U.T.) by opaque envelopes.

All patients were scheduled between 9 and 11 am to avoid any variations in the anxiety and salivary cortisol levels due to circadian rhythms. One patient from the test group did not show up at the surgery appointment. Therefore, the data from 41 participants was analyzed.

### Mindfulness meditation group

Each participant assigned to the MM group individually received MM training for 20 min daily for 3 days before surgery^48^. Training was conducted by one expert periodontist who is also an internationally certified MBSR instructor (O.U.T.). In session 1, the participant sat in a chair, and was instructed to close his/her eyes and relax and to focus on the flow of his/her breath. If a random thought arose, the patient was told to passively notice and acknowledge that thought and to simply let ‘‘it’’ go, by bringing attention back to the sensations of the breath. The last 7 min of day 1 were held in silence, so that the participant could effectively practice mindfulness meditation. In session 2, the facilitator instructed participants to focus on the ‘‘full breath,’’ (sensations in the nostrils and abdomen) and the last 7 min of the session 2 were held in silence. Session 3 was an extension of sessions 1 and 2^48^. As a manipulation check, each subject was asked ‘‘if they felt that they were truly meditating’’ after each meditation session.

### Interventions

On the day of the surgery, all patients in the test and control groups were instructed to arrive 20 min earlier than the surgery appointment hour in order to complete the forms (STAI-S, STAI-T, MDAS) and to record the baseline measurements (HR, SBP, DBP, CL, BIS). This period was standardized at 20 min for each participant. After the first 20 min of the pre-operative measurement period, the control group was taken into operation and the test group was taken into meditation.

The participants of the test group were further informed about MM and they were reassured that the meditation would not have any direct impact on the surgical procedure and that they could discontinue the session at any given point. They were subjected to MM in a room specially allocated for study purposes. Care was taken to ensure that no disturbance occurred or that any third person entered the room throughout the intervention. Shortened guided body scan meditation adapted from the MBSR program by Jon Kabat-Zinn^21^ was performed for 20 min (by O.U.T.). Participants were instructed to focus their awareness on sound guided by the meditator and sensations felt by the body, and merely observe non-judgmentally for any sensations, emotions, or thoughts that may arise during the meditation practice.

### Dental implant surgery

One week before surgery, all patients received an explanation about the dental implant procedure, which lasted for 20 min. Implant placement was performed under infiltration local anesthesia (4% articaine with 1:100,000 adrenaline). All patients received only one carpule of the anesthetic solution immediately at the beginning of the procedure. This amount was sufficient for all patients and no further anesthesia was needed. Surgeries were performed according to the standard single implant placement guidelines^49^ by an expert periodontist (F.D.).

### Data collection and outcome measures

#### Bispectral index (BIS) values and hemodynamic parameters

The primary outcome of the study was the level of sedation achieved by MM, which was assessed with BIS device (Covidien llc Mansfield, MA USA). BIS index, which provides a quantitative measure of sedation to analyze the sedation effect of MM in patients, was used before and during surgery^16,18,19^. The BIS values range between 100 and 0 and give clinicians objective information about the depth of sedation. General anesthesia comprises values ranging of 40 to 60, while deep sedation is within 60 to 70 and 70 to 90 represents light to moderate sedation. The patient is considered awake for values over 90 and adequately sedated consciously for a BIS range of 80 to 85 for oral and maxillofacial surgeries^15,17^.

The BIS electrodes were placed at the attendance of the patient together with monitorization for systolic (SBP) and diastolic blood pressure (DBP), arterial blood oxygen saturation (SpO_2_) and heart rate value (HR) (GE Medical Systems, Freiburg, Germany) at baseline. All values were recorded at 5-min intervals:

- During 20 min of preparation time (in which the preoperative forms were completed) for both groups;
- During MM for the test group
- During surgery for both groups

and at the completion of the surgery.

#### Saliva sample analysis

In order to evaluate cortisol levels (CL), saliva samples were collected at each STAI completion phase. Prior to the collection of saliva, subjects were asked to rinse their mouths with plain water to clear away any debris or food. Care was taken to ensure the non-contamination of saliva with blood.

Salivary cortisol was estimated by solid phase enzyme-linked immunosorbent assay (ELISA) (DiaMetra, Boldon, UK) in ng/ml. The procedure was done as per the manufacturer guidelines on saliva samples.

#### Anxiety

The validated Turkish version of State-Trait Anxiety Inventory (STAI) form^50^ was used to assess the self-reported anxiety levels immediately at the attendance of the patient in the clinic (T0), immediately before (T1) and after (T3) surgery for both groups. STAI comprises separate self-report scales for measuring two distinct anxiety concepts: state anxiety and trait anxiety. The STAI-T scale consists of 20 statements that ask people to describe how they generally feel. The STAI-S scale also consists of an additional 20 statements, but the instructions require subjects to indicate how they feel at a particular moment in time. The STAI-S scale can be used to determine the actual levels of anxiety intensity induced by stressful procedures. Each question is rated on a 4-point scale (not at all, somewhat, moderately so, very much so). The range of possible scores is between 20 and 80. STAI scores are commonly classified as “no or low anxiety” (20–37), “moderate anxiety” (38–44), and “high anxiety” (45–80).

Subjective well-being was assessed (with the affect balance scale of a five-point answering scale ranging from 1 = ‘‘low well-being’’ to 5 = ‘‘high well-being’’). In addition, the education level of the participants was recorded (1 = secondary education, 2 = tertiary education, 3 = Bachelors degree)^51^.

MDAS is a modification of Corah’s Dental Anxiety Scale consists of 5 questions with scores ranging from 0 (not anxious) to 5 (extremely anxious) for each answer for a total score ranging between 0 and 25. The score of 0 to 5 is considered not anxious, 6 to 10 is low anxiety, 11 to 14 is moderate anxiety, 15 to 18 is high anxiety and 19 to 25 is extreme anxiety/phobic.

Three different periodontists who did not participate in the procedure; checked the completion of the forms (STAI-S, STAI-T, MDAS) (M.C.H.), took the salivary samples (B.A.) and recorded the vital signs (HR, SpO_2_, SBP, DBP) and BIS monitoring data (M.O.).

#### Sample size

The study was conducted with a small effect size of 0.25 and sample size was calculated with G*Power 3.1.9.7 for 4 repeated measures in 2 study groups. For the sample size calculation of within-between interaction ANOVA, power of the study was set at 95% and type I error was taken as 5%. Minimum of 36 subjects would be necessary for the study, however as dropouts are very common in studies, we increased the estimated sample 20%. The needed sample size was 42 (21 in each group).

#### Statistical analysis

Categorical variables were expressed as numbers and percentages, and Chi Square test was used to compare the groups. Continuous variables were summarized as mean and standard deviation and as median and quartiles (Q1–Q3) where appropriate. The normality of the distribution for continuous variables was confirmed with the Kolmogorov–Smirnov test. For comparison of continuous variables between two groups, Mann–Whitney U test was used and Friedman test was used for time-dependent measures. Spearman correlation test was used between 2 continuous variables. The F1-LD-F1 design was used for longitudinal data analysis because the data were not normally distributed. The F1-LD-F1 model generates an ANOVA-type statistic for group, time, and group-time interaction. The F1-LD-F1 model, in addition to the ANOVA-type statistic, provides relative treatment effects (RTE) as a unitless measure of effect size. The RTE has values ranging from 0 to 1 and can be interpreted as follows: An RTE of 0.25 for a specific subgroup indicates that the probability of a randomly selected person from this subgroup outperforming a randomly selected person from the entire dataset is estimated to be 25%. The probability of a randomly selected person from this subgroup scoring lower than a randomly selected person from the entire dataset, on the other hand, is estimated to be 75%. An RTE of 0.50 indicates that there is no tendency for a higher or lower score in any subgroup. All statistical analyses were carried out using IBM SPSS 20 (Armonk, NY: IBM Corp.) and RStudio (RStudio Team, 2020). The npar LD package was used in RStudio for the F1-LD-F1 design. The statistical level of significance for all tests was considered to be 0.05.

## Results

**Figure 1:**
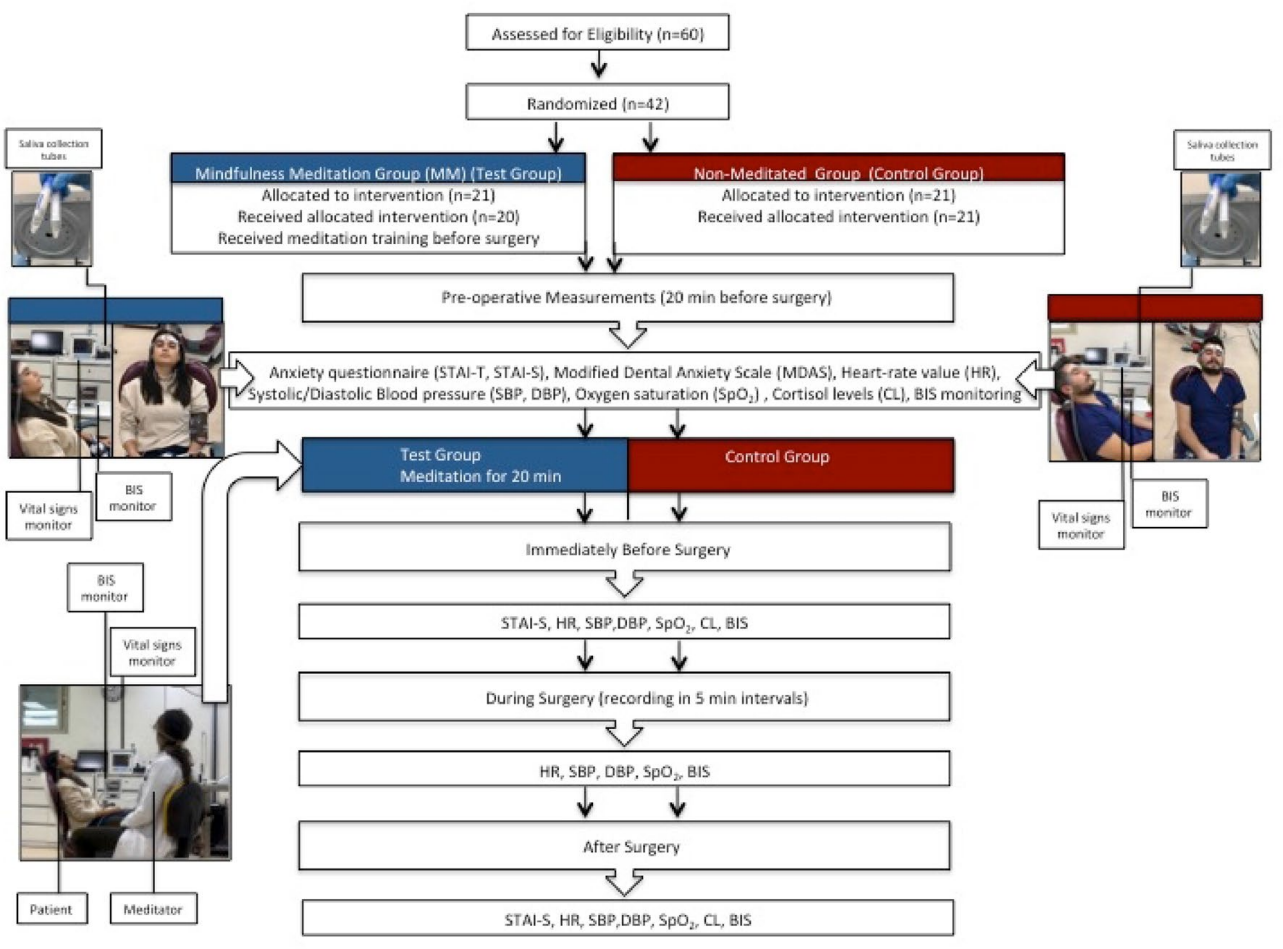
A flow diagram showing the enrollment, allocation and analysis of subjects involved in the RCT.

A total of 60 participants were screened and 42 eligible patients were scheduled for the surgery. Of these participants, 21 patients were randomized each for the test (MM) and control group (Fig. 1).

The socio-demographic details of participants are shown in Table 1. Test and control groups were similar in gender, age, education and well-being scale parameters at the baseline.

**Table 1:** Baseline socio-demographic characteristics of the participants (n = 41).

		Control		Test (MM)		*p *value^+^
		N	(%)	N	(%)	
Gender	Male	9	(45.0)	8	(38.1)	0.654
	Female	11	(55.0)	13	(61.9)	
Age	25–44	11	(55.0)	14	(66.7)	0.444
	45–66	9	(45.0)	7	(33.3)	
Education	Tertiary education	14	(70.0)	13	(61.9)	0.585
	Bachelors degree	6	(30.0)	8	(38.1)	
Well-being scale	2	11	(55.0)	12	(57.1)	0.890
	3	9	(45.0)	9	(42.9)	

*p* value^+^; Chi square test.

**Figure 2:**
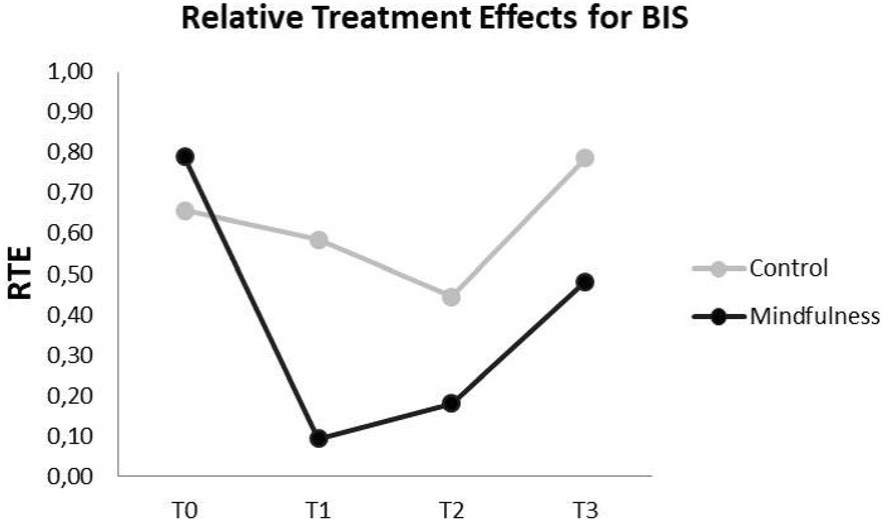
The Relative treatment effects for BIS.

The results of the study are summarized in Tables 2, 3 and 4. The BIS values showed significant differences among study groups and over time. (Tables 3 and 4, Fig. 2). While the BIS values were similar at baseline (T0) in both groups, there was a significant decrease in BIS in the MM group with no changes in the controls at (T1). At the time of the surgery (T2), the BIS values decreased slightly in the control group compared to the baseline, while they were still considerably lower than the baseline value in the MM group. After the operation (T3), the BIS value increased in both groups. However, while the BIS value even exceeded the baseline measurements in the control group, it was still lower than the baseline measurement in the MM group (Fig. 2).

**Table 2:** Comparison of hemodynamic parameters (HR, SBP, DBP, SpO_2_), bispectral index score and cortisol levels at baseline (T0), immediately before surgery (T1), during surgery (T2) and immediately after surgery (T3) between test and control groups.

		Control		Test (MM)		*p *value^+^
		Mean ± SD	Median (25th–75th)	Mean ± SD	Median (25th–75th)	
HR	T0	83.7 ± 8.4^a,b^	80.5 (77.5–89.5)	84.9 ± 8.7^a,b,c^	82.0 (80.0–93.0)	0.539
	T1	89.3 ± 10.2^c^	85.0 (81.5–97.0)	73.3 ± 9.4^b,c^	73.0 (65.0–79.0)	** < 0.001**
	T2	91.0 ± 8.0^c^	88.0 (85.0–94.8)	78.7 ± 9.1^c^	79.0 (72.0–86.0)	** < 0.001**
	T3	82.6 ± 7.7	81.0 (78.5–87.5)	76.3 ± 7.2	75.0 (70.0–81.0)	0.025
	*p *value^++^	** < 0.001**		** < 0.001**		
DBP	T0	76.4 ± 11.7	72.5 (70.5–80.0)	74.2 ± 10.9	76.0 (68.0–83.0)	0.865
	T1	80.8 ± 12.3	79.5 (72.0–85.0)	71.8 ± 9.3	72.0 (65.0–77.0)	**0.026**
	T2	79.8 ± 7.4^c^	80.3 (73.3–86.3)	75.0 ± 9.0	76.5 (70.0–81.0)	0.148
	T3	74.4 ± 6.3	73.0 (69.0–79.5)	75.5 ± 7.9	74.0 (70.0–82.0)	0.480
	*p *value^++^	**0.005**		NS		
SBP	T0	135.5 ± 22.7^a,b^	127.5 (121.0–149.0)	130.9 ± 19.5^a^	130.0 (116.0–141.0)	0.705
	T1	144.1 ± 23.4^c^	143.0 (127.5–156.0)	122.8 ± 13.7^b,c^	126.0 (114–131)	**0.003**
	T2	145.3 ± 19.8^c^	143.3 (131.0–153.0)	131.1 ± 16.7	135.5 (119.5–143.5)	0.050
	T3	136.0 ± 18.1	130.0 (124.5–146.0)	129.9 ± 17.1	135.0 (118–140)	0.657
	*p *value^++^	** < 0.001**		** < 0.001**		
SpO_2_	T0	96.7 ± 1.0^a^	97.0 (96.0–97.0)	96.5 ± 1.4^a,b,c^	96.0 (96.0–98.0)	0.745
	T1	95.5 ± 1.0	95.0 (95.0–96.0)	98.2 ± 1.1	98.0 (98.0–99.0)	** < 0.001**
	T2	96.1 ± 0.6	96.0 (95.5–96.5)	97.7 ± 0.9	97.5 (97.0–98.5)	** < 0.001**
	T3	96.4 ± 1.5	96.0 (96.0–97.5)	97.8 ± 1.5	98.0 (98.0–98.0)	**0.005**
	*p *value^++^	** < 0.001**		** < 0.001**		
BIS	T0	97.3 ± 0.6	97.0 (97.0–98.0)	97.7 ± 0.5^a,b^	98.0 (97.0–98.0)	**0.014**
	T1	97.0 ± 0.6	97.0 (97.0–97.0)	76.2 ± 9.6^c^	79.0 (73.0–80.0)	** < 0.001**
	T2	96.6 ± 0.5	96.5 (96.3–97.0)	85.7 ± 4.0^c^	86.0 (85.0–87.5)	** < 0.001**
	T3	97.7 ± 0.5	98.0 (97.0–98.0)	96.2 ± 1.4	97.0 (96.0–97.0)	** < 0.001**
	*p *value^++^	NS		** < 0.001**		
CL	T0	34.7 ± 5.4^a^	35.1 (31.3–38.7)	36.0 ± 6.7^a,c^	37.1 (30.8–41.5)	0.611
	T1	40.6 ± 5.7^c^	40.3 (36.0–43.9)	29.2 ± 6.9	29.4 (24.3–34.4)	** < 0.001**
	T3	35.9 ± 5.0	35.4 (32.5–38.1)	27.8 ± 6.9	29.4 (24.6–32.0)	** < 0.001**
	*p *value^++^	** < 0.001**		** < 0.001**		

Significant values are in bold.

*HR* Heart rate (BPM), *DBP* Diastolic blood pressure (mmHg), *SBP* Systolic blood pressure (mmHg), *SpO*_*2*_ Oxygen saturation (%), *BIS* Bispectral index score, *CL* Cortisol level (µg/dL), *SD* Standard deviation (*p* < 0.05). *p* value^+^ ; Mann–Whitney U test, *p* value^++^; Friedman test within groups between times.

^a^Between T1 *p* < 0.05.

^b^Between T2 *p* < 0.05.

^c^Between T3 *p* < 0.05.

**Table 3:** Comparison of anxiety levels (M-DAS, STAI-S, STAI-T) at the baseline (T0), immediately before surgery (T1) and immediately after surgery (T3) between test and control groups.

		Control		Test (MM)		*p *value^+^
		Mean ± SD	Median (25th–75th)	Mean ± SD	Median (25th–75th)	
MDAS	T0	12.9 ± 2.8	13.0 (11.5–14.5)	12.7 ± 4.0	12.0 (9.0–16.0)	0.386
STAI-T	T0	49.6 ± 4.9	50.0 (45.0–53.5)	50.3 ± 7.8	49.0 (46.0–55.0)	0.704
STAI-S	T0	48.7 ± 4.7^a,c^	47.5 (45.0–51.0)	47.3 ± 7.6^a,c^	47.0 (43.0–51.0)	0.522
	T1	59.0 ± 5.4^c^	58.5 (56.5–61.0)	25.4 ± 4.9	23.0 (23.0–27.0)	** < 0.001**
	T3	39.1 ± 5.0	39.0 (35.5–42.5)	25.8 ± 5.1	24.0 (22.0–27.0)	** < 0.001**
	*p *value^++^	** < 0.001**		** < 0.001**		

Significant values are in bold.

*M-DAS* Modification of Corah’s dental anxiety scale, *STAI-T* State-trait anxiety inventory-trait version, *STAI-S* State-trait anxiety inventory-state version. *p* value^+^; Mann–Whitney U test, *p* value^++^; Friedman test within groups between times.

^a^Between T1 *p* < 0.05.

^c^Between T3 *p* < 0.05.

**Table 4:** Results of F1-LD-F1 model and relative treatment effects.

	F1-LD-F1 results			Relative treatment effects (RTE)					
		F(*df)*	*p* value	Time		Control		Mindfulness (MM)	
HR	Group	12.14(1)	< 0.001	T0	0.55	Cont*T0	0.53	MM*T0	0.57
	Time	13.35(3)	< 0.001	T1	0.46	Cont*T1	0.68	MM*T1	0.25
	Group*Time	24.31(3)	< 0.001	T2	0.58	Cont*T2	0.77	MM*T2	0.40
				T3	0.41	Cont*T3	0.50	MM*T3	0.32
SBP	Group	2.75(1)	0.096	T0	0.47	Cont*T0	0.49	MM*T0	0.44
	Time	8.87(3)	< 0.001	T1	0.47	Cont*T1	0.62	MM*T1	0.33
	Group*Time	12.46(3)	< 0.001	T2	0.58	Cont*T2	0.66	MM*T2	0.50
				T3	0.49	Cont*T3	0.51	MM*T3	0.47
DBP	Group	0.79(1)	0.372	T0	0.48	Cont*T0	0.48	MM*T0	0.48
	Time	3.24(3)	0.036	T1	0.50	Cont*T1	0.59	MM*T1	0.39
	Group*Time	6.88(3)	< 0.001	T2	0.56	Cont*T2	0.63	MM*T2	0.49
				T3	0.47	Cont*T3	0.44	MM*T3	0.50
SpO_2_	Group	41.6(1)	< 0.001	T0	0.45	Cont*T0	0.46	MM*T0	0.44
	Time	1.63(3)	0.186	T1	0.49	Cont*T1	0.21	MM*T1	0.77
	Group*Time	14.92(3)	< 0.001	T2	0.50	Cont*T2	0.32	MM*T2	0.67
				T3	0.55	Cont*T3	0.41	MM*T3	0.68
BIS	Group	58.41(1)	< 0.001	T0	0.72	Cont*T0	0.66	MM*T0	0.79
	Time	118.56(3)	< 0.001	T1	0.34	Cont*T1	0.59	MM*T1	0.09
	Group*Time	47.75(3)	< 0.001	T2	0.31	Cont*T2	0.45	MM*T2	0.18
				T3	0.63	Cont*T3	0.79	MM*T3	0.48
CL	Group	12.16(1)	< 0.001	T0	0.56	Cont*T0	0.54	MM*T0	0.58
	Time	13.31(2)	< 0.001	T1	0.53	Cont*T1	0.75	MM*T1	0.31
	Group*Time	38.59(2)	< 0.001	T2	0.42	Cont*T2	0.57	MM*T2	0.27
STAI-S	Group	248.78(1)	< 0.001	T0	0.67	Cont*T0	0.68	MM*T0	0.65
	Time	82.23(2)	< 0.001	T1	0.53	Cont*T1	0.89	MM*T1	0.18
	Group*Time	77.96(2)	< 0.001	T2	0.31	Cont*T2	0.44	MM*T2	0.19

*SBP* Systolic blood pressure, *DBP* Diastolic blood pressure, *SpO*_*2*_ Arterial blood oxygen saturation, *BIS* Bispectral index, *CL* Cortisol level, *STAI-S* State-trait anxiety inventory-state version, *RTE* Relative treatment effect, *df* Degrees of freedom. RTE value gives the probability that the measurement level of the variable is higher than the overall average. If the RTE is low, the value can be interpreted as low, and if it is high, the value can be interpreted as high. If it is around 50%, it means that there is no significant effect.

**Figure 3:**
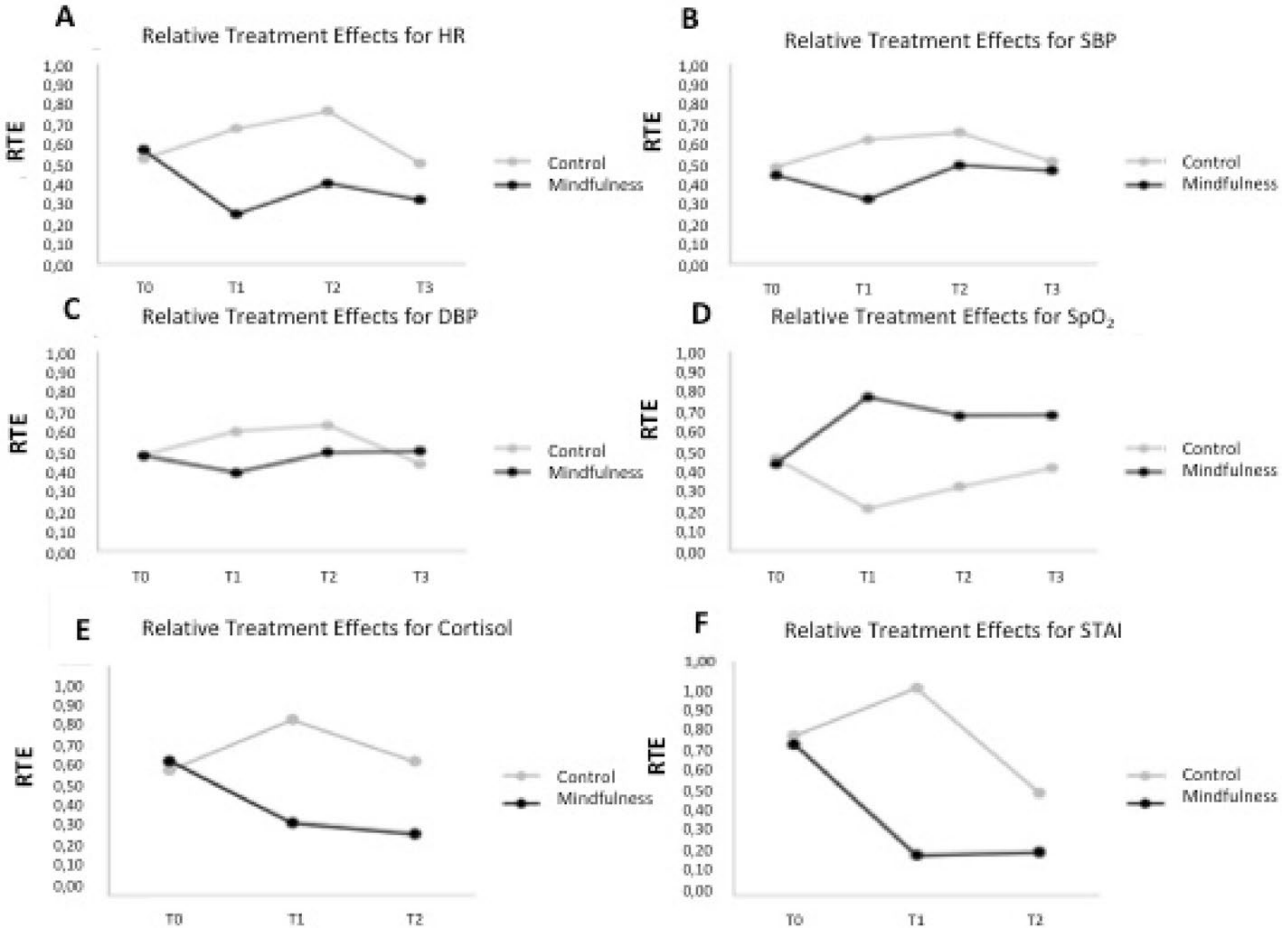
The Relative treatment effects for HR (**A**), SBP (**B**), DBP (**C**), SpO_2_ (**D**), Cortisol (**E**) and STAI-S (**F**).

While the baseline values of all hemodynamic parameters were similar at baseline, the patients in the test group had significantly lower HR (BPM) after MM (T1) and this reduction lasted during and after surgery (Fig. 3A). The DSP (mmHg) and SBP (mmHg) were also significantly lower in the test group after MM while SpO_2_ (%) levels were higher at all evaluation time periods in this group. Although the group effect was not significant for overall in SBP and DBP, time and group*time interaction were statistically significant (Table 4). (Fig. 3B and C). The time-dependent change for oxygen saturation was not statistically significant, but the group and time*group interaction was significant (Table 4).

While the saturation level increased at (T1) in the MM group, it decreased in the controls. The saturation levels stayed higher during and after surgery in the MM group (Fig. 3D).

The cortisol levels and STAI were in accordance with the BIS values showing a significant decrease after meditation and after surgery in the MM group and the change over time was statistically significant (Table 4). The control group had a significant increase in cortisol (µg/dL) and STAI levels even after the operation (Fig. 3E,F). The correlation analysis showed that the STAI scores had a significant positive correlation with pulse (r = 0.35) and with CL (r = 0.5) while a negative correlation was found with SpO2 (r = − 0.44) at T3.

Overall, the statistical relative treatment effect analysis showed that the MM had significant effects on BIS, Cortisol, STAI, SBP, DBP, SpO_2_ and HR.

## Discussion

This is the first randomized controlled clinical study that demonstrates the efficacy of a 20-min mindfulness meditation intervention in rapidly reducing the surgery anxiety of patients during dental implant operations. The findings suggest that the mindfulness meditation had significant positive effects on cardiovascular parameters, physiological findings and biochemical outcomes.

Dental implant surgery under local anesthesia comprises a major part of oral surgery. Although local anesthesia usually provides adequate analgesia for surgery, patients usually suffer from the discomfort and pain associated with surgical procedures^15^. Anxiety toward surgical procedures is usually associated with a suppressed fear of pain. These patients not only find these procedures unpleasant but they may also exhibit peripheral manifestations such as sympathetic overactivity.

Mindfulness as a state, trait, and clinical intervention has been extensively researched over the last two decades^22,52,53^. The recent meta-analysis of RCTs demonstrated that mindfulness meditation is associated with reduced physiological markers of stress^22,52^ such as decreased HR^40^ decreased BP^41^ and decreased cortisol levels^34^. To this extent, we examined whether 20 min of mindfulness meditation would improve physiological variables i.e., cardiovascular parameters (HR, SBP, DBP, SpO_2_). The results we present are encouraging in that they suggest certain immediate physiological effects of MM and that meditation may have an effect on decreased activation of the SNS.

Previous studies have demonstrated reductions in cortisol subsequent to mindfulness interventions^54,55^. Similar to the findings of previous research, this study showed that the patients in the test group had significantly lower cortisol levels immediately after MM and after surgery. A recent systematic review highlights some potential causes of inconsistent and contrary findings of mindfulness effects interventions on cortisol levels such as; research design (lack of controlled trials), sampling time and protocols, cortisol measurement (baseline effects on cortisol), participation standardization (effects of different subgroups)^43^. In this randomized controlled clinical trial, cortisol samples were collected at the same time of the day and analyzed by the same Elisa protocol in terms of standardization.

Growing evidence has indicated that mindfulness practice induces both state and trait changes; that is, it temporarily changes the condition of the brain and the corresponding pattern of activity or connectivity^53^. Yao et al.^44^ used the STAI and suggested that mindfulness training may potentially help alleviate patients’ dental anxiety. Similarly, we evaluated psychological outcomes using the STAI-S. MM was found to be effective in significantly reducing the STAI-S when compared to the control group before and after surgery, suggesting that MM can provide both immediate and lasting effects on anxiety during dental surgery.

Taniyama et al.^17^ found that the BIS gradually decreased during initial medicated sedative loading and ranged between 80 and 85 at the time of optimal sedation, which is similar to our findings in the test group. In our study, there was significant difference in the recorded bispectral analysis values between two groups in two time periods (immediately before and during surgery). It was observed that the BIS value gradually decreased during MM in the test group (to 73- 80). However, when surgery was started, various stimuli were added, which affected patients’ sedation levels. Therefore, the BIS values tended to increase during surgery (85 to 87.5). At the optimal sedation level, BIS was significantly lower than pre-surgery (97) and during surgery values compared to the control group (96.3–97). The significant decrease of BIS values in the MM group (which resembles the optimal condition for medical sedation) may be related to a diminished activation of the amygdala in response to emotional stimuli during mindful states as well as in a resting state, suggesting increased parasympathetic tone and decreased sympathetic activity^56,57^.

We provided a brief mindfulness training for the MM group. According to Zeidan et al.^40^, the advantages of the mindfulness meditation technique can be experienced immediately after a short training program^58^. One of the most intriguing findings in the field of brain structure and meditation is the minimal number of hours of meditation practice needed to induce neuroplastic changes^22,59,60^. These studies suggest that even short-term meditation practice can involve and modify many of the same brain regions that exhibit variations in long-term practitioners. Early-onset structural changes may endure following initial meditation practice, be maintained through ongoing practice, or result from a combination of both. However it has been reported that the response to MM variess significantly among individuals within the range of minutes to months. Although a training program was conducted for the participants of the MM group, there was no data regarding their level of MM experience. This may effect the outcomes of this randomized controlled study^22,53,61^.

Tang^53^ and Hölzel^22^ have reported that: “practitioners could have pre-existing differences… which might be linked to their interest in meditation, personality.” This highlights a notable confound in previous research on mindfulness literature i.e., the MM instructors’ individualistic disparities, such as the educational level and experience of the meditator. In the current study, the brief body scan meditation was performed by an MBSR instructor with 10 years of experience. In addition, individual differences in personality are likely to contribute to how people respond to and benefit from mindfulness practice, similar to the differences in brain function and structure, genetic predisposition, life experiences and environmental factors. In order to overcome this factor, the test and the control group were matched for age, sex, socio-economical status and educational level.

This study has some limitations. First, the study was conducted in a single center with a relatively small sample size. Second, anxiety and hemodynamic parameters are complex multicausal conditions; thus, there may well be more confounding factors that explain the variance in anxiety and hemodynamic parameters (such as age, gender and systemic health) than those found in this study. Although a large number of studies have used hemodynamic parameters in the physiological assessment of anxiety, it should be taken into account that there are also a few studies with contradictory results in this regard. Third, the local anesthesia during the procedure might affect hemodynamic parameters, which could have potential consequences for both physiological and psychological status.

In addition to these limitations, the patients in the control group knew that they would not receive pre-operative meditation, which may potentially affect their anxiety levels. As the patients of the control group were immediately taken to surgery after T1 while the patients of the test group received MM after T1, this additional time period may introduce a confounder factor for the outcomes. Furthermore, MM was performed by another dentist other than the operator in the current study. It has been reported that the dentist-patient relationship is another important issue, which is the core of dentistry. A good therapeutic alliance during dental therapy, including adequate, individualized, and nonjudgmental education, is of the utmost importance in order to decrease the patient's negative emotions, allow the patient to gain insight about the disease condition, and increase their feeling of control over the situation^62,63^. Successful relationships require physicians to practice a welcoming stance, participatory decision-making, and mindfulness about both the patient's and the dentist's inner lives^64^. It was also reported that dental clinics are encouraged to incorporate mindfulness interventions into their routine daily practice^44^. The results of this study also highlighted that mindfulness is not only associated with a reduction of general anxiety, but also with dental anxiety specifically. These findings show that clinical practitioners treating dental anxiety provide more emotional comfort for patients. Therefore, it may be suggested that if the dentist who is actively carrying out the surgical procedure also directly performs the MM, this may eventually result in better outcomes.

As compared with the non-meditated group, mindfulness meditation appeared to be a feasible and reliable strategy for managing stress during dental implant operations with benefits in psychological, physiological and biochemical outcomes. Overall, the results provide evidence that brief mindfulness meditation is effective for reducing dental anxiety. We believe that these findings will provide valuable evidence to guide future mindfulness-based intervention research in the dental field.

### Supplementary Information

Supplementary Information.
